# Identification and classification of host cell proteins during biopharmaceutical process development

**DOI:** 10.1002/btpr.3224

**Published:** 2021-11-18

**Authors:** Louisa J. Wilson, Will Lewis, Richard Kucia‐Tran, Daniel G. Bracewell

**Affiliations:** ^1^ The Advanced Centre for Biochemical Engineering, Department of Biochemical Engineering University College London London UK; ^2^ GlaxoSmithKline Stevenage Hertfordshire UK

**Keywords:** downstream processing, host cell proteins, mass spectrometry, monoclonal antibodies, product quality, upstream processing

## Abstract

As significant improvements in volumetric antibody productivity have been achieved by advances in upstream processing over the last decade, and harvest material has become progressively more difficult to recover with these intensified upstream operations, the segregation of upstream and downstream processing has remained largely unchanged. By integrating upstream and downstream process development, product purification issues are given consideration during the optimization of upstream operating conditions, which mitigates the need for extensive and expensive clearance strategies downstream. To investigate the impact of cell culture duration on critical quality attributes, CHO‐expressed IgG1 was cultivated in two 2 L bioreactors with samples taken on days 8, 10, 13, 15, and 17. The material was centrifuged, filtered and protein A purified on a 1 ml HiTrap column. Host cell protein (HCP) identification by mass spectrometry (MS) was applied to this system to provide insights into cellular behavior and HCP carryover during protein A purification. It was shown that as cultivation progressed from day 8 to 17 and antibody titer increased, product quality declined due to an increase in post‐protein A HCPs (from 72 to 475 peptides detected by MS) and a decrease in product monomer percentage (from 98% to 95.5%). Additionally, the MS data revealed an increase in the abundance of several classes of post‐protein A HCPs (e.g., stress response proteins and indicators of cell age), particularly on days 15 and 17 of culture, which were associated with significant increases in total overall HCP levels. This provides new insight into the specific types of HCPs that are retained during mAb purification and may be used to aid process development strategies.

## 1 INTRODUCTION

The host cells that are used for the expression of mAbs, produce not only the desired product, but also co‐express the endogenous proteins that enable the cells to live and grow. These so‐called host cell proteins (HCPs) are present in the harvested cell culture fluid (HCCF) and require separation from the mAb product during downstream processing.

HCPs are a complex mixture of various proteins with significantly diverse physicochemical properties^1^, ^2^ requiring the use of several techniques for their efficient removal. HCP clearance is crucial as their presence can influence drug efficacy and cause immunogenic responses in patients, including cross‐reactivity and autoimmunity.^2^, ^3^, ^4^, ^5^, ^6^ The United States Food and Drug Administration (FDA) suggests HCPs be reduced to acceptably low levels (<100 ppm),^5^, ^7^ although in reality HCP limits are case‐by‐case dependent and are defined from (pre‐) clinical studies and manufacturing consistency lots.^8^, ^9^ The recommended limit is only a guideline and is aimed at ensuring the level of impurities is reduced as much as possible, since limited understanding of the exact types of HCP species that are being retained in the final drug product means it is unclear how dangerous their presence may be to the patient. Low levels of HCPs overall reduce the possibility that harmful types of HCP species are still present in the final drug substance and pose a risk to patients.

Several research groups have demonstrated that most HCPs associated with mAbs after protein A affinity chromatography are co‐eluting with the product through association with the bound antibodies rather than by nonspecifically binding to the resin.^2^, ^10^, ^11^, ^12^, ^13^, ^14^ Based on this understanding, considerable research has been done to identify the specific HCP species that are being retained during protein A affinity chromatography with certain antibodies expressed in CHO cells (Table 1). HCPs reported to be present in high amounts include those that are involved in essential cell survival processes such as in translation (e.g., elongation factor 2), in protein folding (e.g., heat‐shock proteins Hsp70 and Hsp90 and clusterin), and in glucose or lipid metabolism (e.g., Glyceraldehyde 3‐phosphate dehydrogenase; pyruvate kinase, lactate dehydrogenase; PLBL2).^15^, ^16^, ^17^, ^18^ In addition, proteases such as cathepsins and serine protease HTRA1 have been identified, particularly during late stages of the culture process when they are suggested to cause protein fragmentation.^15^, ^16^, ^17^, ^18^, ^19^

**TABLE 1:** Selection of HCP species that co‐elute with monoclonal antibodies during protein A purification, as identified in various literature

Identified post‐protein A HCPs	Source
78 kDa glucose‐regulated protein	Farrell (2015),^17^ Zhang (2014),^16^ and Zhang (2016)^27^
Actin cytoplasmic 1	Farrell (2015),^17^ Zhang (2014),^16^ and Zhang (2016)^27^
Clusterin	Farrell (2015),^17^ Zhang (2014),^16^ and Zhang (2016)^27^
Elongation factor 1‐alpha 1	Zhang (2014)^16^ and Zhang (2016)^27^
Elongation factor 2	Albrecht (2018),^18^ Tait (2012),^15^ Zhang (2014),^16^ and Zhang (2016)^27^
Glutathione S‐transferase P	Albrecht (2018)^18^ and Zhang (2016)^27^
Glyceraldehyde‐3‐phosphate dehydrogenase	Albrecht (2018),^18^ Farrell (2015),^17^ and Zhang (2016)^27^
Heat shock cognate 71 kDa protein	Albrecht (2018)^18^ and Zhang (2016)^27^
Peptidyl‐prolyl cis‐trans isomerase	Albrecht (2018),^18^ Tait (2012),^15^ and Zhang (2016)^27^
Peroxiredoxin‐1	Albrecht (2018),^18^ Farrell (2015),^17^ and Zhang (2016)^27^
Phosphoglycerate kinase 1	Zhang (2016)^27^
Pyruvate kinase	Tait (2012),^15^ Zhang (2014),^16^ and Zhang (2016)^27^
Serine protease HTRA1	Farrell (2015)^17^ and Zhang (2016)^27^

Abbreviation: HCPs, host cell proteins.

Research into the effects of upstream operating conditions on HCP profiles of unprocessed cell culture material has also been carried out. Jin et al.^1^ investigated the impact of media, temperature, feeding strategy, agitation speed, process duration and cell viability on composition of HCPs and found viability to have the most significant effect. Not only did they measure higher levels of HCPs on day 15 of culture—when viability was only 11%—but they also discovered that low‐molecular weight species were more abundant at this time in the culture process, suggesting the release of proteases and the associated degradation of proteins at low viability. In other work, changes in HCP profiles over the course of a cell culture were demonstrated to be due to associated changes in environment, metabolism, and declining viability.^15^ Both these findings highlight the importance of controlling cell culture duration and cell viability and show that time of harvest is a crucial parameter with regards to HCP composition.

However, to date, only a few published works have linked upstream and downstream studies together.^16^, ^17^, ^20^, ^21^ In a previous paper,^22^ we have shown that upstream operating conditions, including culture duration, that are optimized for higher levels of antibody expression, can result in decreased product quality. We have shown the effects of culture duration and upstream harvest time on antibody titer, as well as on product monomer levels and the amount of HCPs present in purified material, which highlighted that culture duration should not be extended purely for the purpose of expressing more antibodies.

In this article, the types of HCP species that are present in processed material from various harvest points are explored to investigate cellular behavior in the context of mAb process development.

## 2 MATERIALS AND METHODS

### 2.1 Cell culture

**FIGURE 1:**
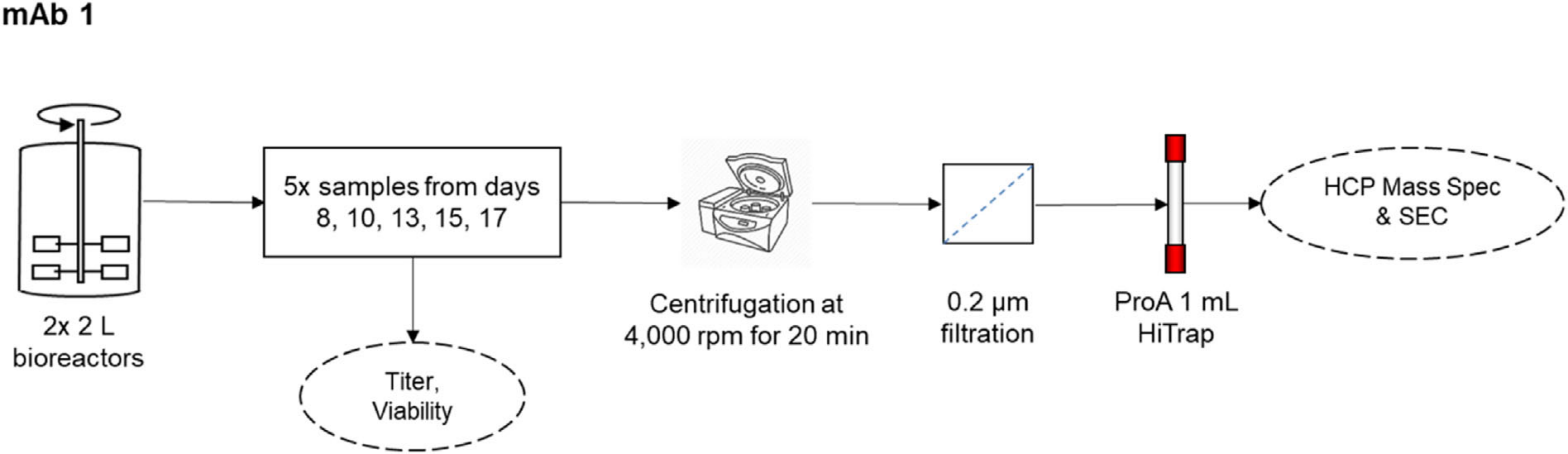
Summary of experimental methods

A summary of the methods is illustrated in Figure 1. CHO‐expressed IgG1 monoclonal antibody (mAb 1) was cultivated in two 2 L bioreactors under fixed culture parameters (batch process with defined temperature, DO, and pH setpoints, and with glucose addition on day 7) using chemically defined media. Samples were taken on days 8, 10, 13, 15, and 17, and culture viability was determined by the trypan blue exclusion method using a benchtop Vi‐Cell XR (Beckman Coulter, Indianapolis, IN). The material was centrifuged in a Sorvall Legend RT (Thermo Scientific, Waltham, MA) at 4000 rpm for 20 min at 4°C. Antibody titer was measured using a CEDEX BioHT (Roche Custom Biotech, Mannheim, Germany). All titer results have been normalized.

### 2.2 Downstream purification

Supernatant samples were filtered using 0.2 μm syringe filters (Mini Kleenpak™ 25 mm syringe filters with 0.2 μm Supor® EKV membrane, Pall Corporation, Portsmouth, UK) to remove cell debris and prepare samples for affinity purification. A 1 ml MabSelect SuRe protein A HiTrap column (Cytiva, Uppsala, Sweden) was used for the purification. The column was equilibrated with a Tris acetate buffer (pH 7.5) before loading to 85% of the manufacturer's suggested dynamic binding capacity at a flowrate of 0.2 ml/min. This was then followed by a column wash step with a Tris acetate buffer containing caprylate (pH 7.5) and a re‐equilibration step before product elution using a sodium acetate buffer (pH 3.6).

### 2.3 Analytical assays

#### 2.3.1 Size exclusion chromatography

The mAb monomer, aggregate and fragment composition of processed samples was determined by size exclusion chromatography (HPLC‐SEC) using an Agilent HPLC system (Agilent 1100 series) and a 7.8 × 300 mm^2^ TSKgel G3000SWXI column (Tosoh Bioscience) with a running buffer containing sodium phosphate (monobasic) and sodium chloride (pH 6.7). The flow rate was 1 ml/min and protein was detected using UV detectors at 214 and 280 nm. The SEC data was analyzed on ChromView for ChemStation version 2.4.2 and has an accuracy of ±0.5% as previously established by GSK's analytical team.

#### 2.3.2 MS for HCP identification

**FIGURE 2:**
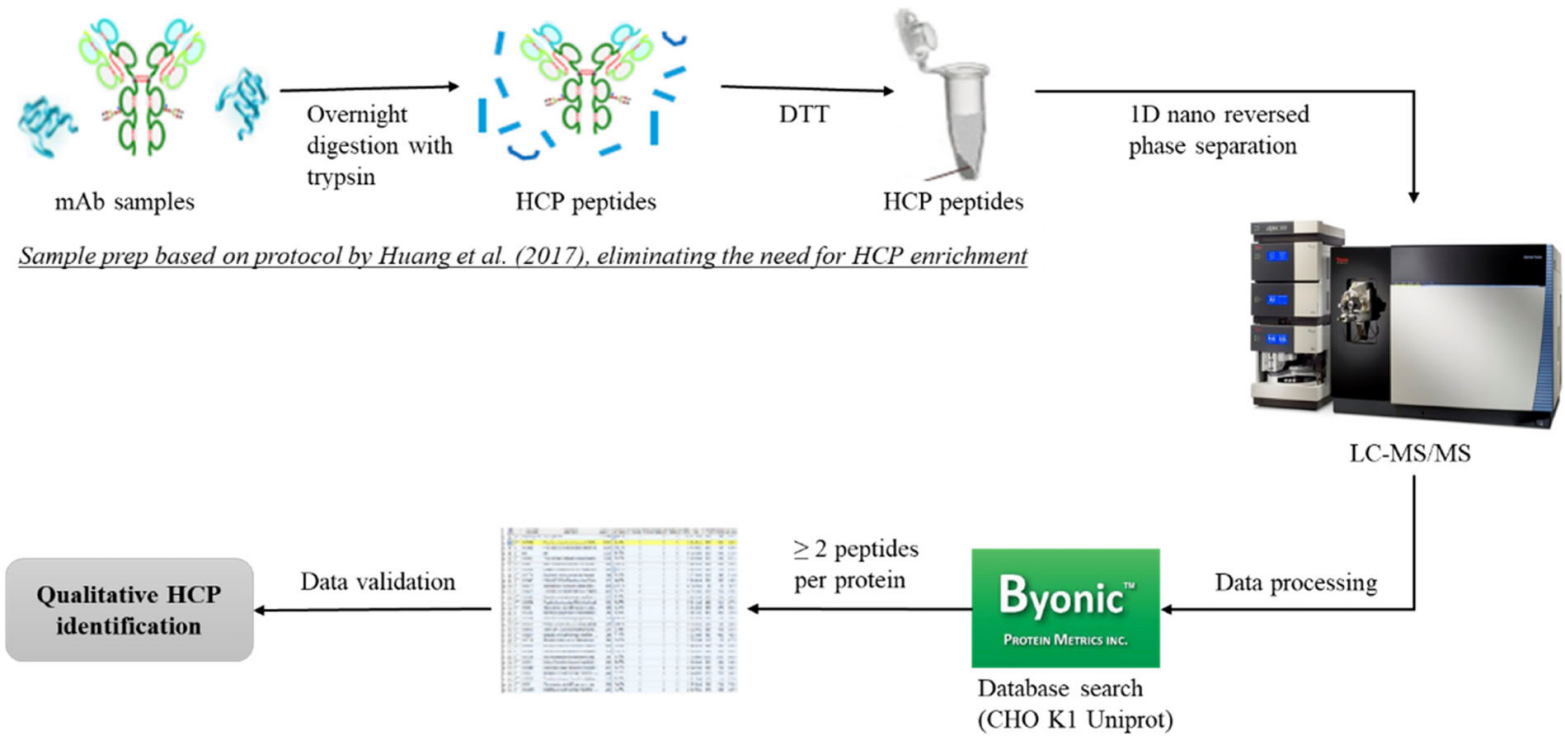
Workflow for HCP identification by mass spectrometry. Figure adapted from Huang et al.^28^ HCP, host cell protein

HCP species present in the processed samples were identified by mass spectrometry (MS) (Figure 2). Samples were prepared by adding 40 μg of protein A purified mAb to 45 μl of 50 mM ammonium bicarbonate and then adding 1 μg/μl trypsin in a 20:1 mAb/trypsin ratio. Samples were incubated overnight at 37°C, and the next day 5 μl of 100 mM DTT were added prior to a further incubation period of 30 min at 37°C. The digestion was stopped by adding 1 μl neat formic acid and then drying the samples by speed‐vacuum. Samples were redissolved in 40 μl 0.1% formic acid and then analyzed on a nano‐LC Orbitrap mass spectrometer.

The HCP‐MS data was processed using the Protein Metrics Byos® Platform. To reduce the risk of false positive results, common contaminants as well as HCPs with only two peptides have been filtered out and a MS/MS score of 150 was applied to accept the MS/MS data quality. A MS/MS (MS2) score is a measure of how well experimental and theoretical peptides match up and is typically used to filter out peptides with poor MS2 fragment coverage. Here, a conservative threshold score of 150 was set based on the recommended settings within the data analysis platform used, providing a good level of confidence that the identified HCPs are indeed present. The remaining data was manually evaluated based on the isotope plot data. Biological process information was obtained for all identified HCP species by searching the UniProt database using protein accession numbers.

## 3 RESULTS AND DISCUSSION

### 3.1 Upstream profiles

**FIGURE 3:**
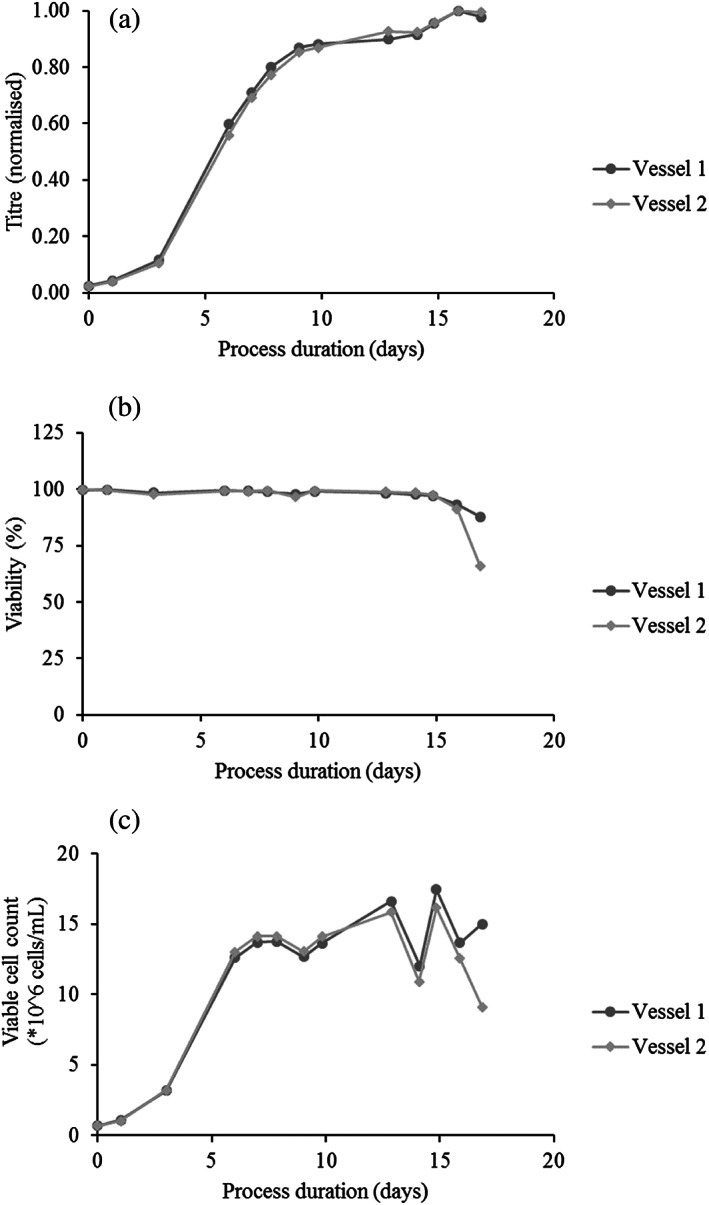
Titer (normalized due to confidentiality), culture viability and viable cell count data for both 2 L bioreactors. Note that the inconsistency in viable cell counts at day 13 is likely due to poor sampling technique as suggested by the rapid restoration of the viable cell count back to the expected range, which was most likely caused by insufficient flushing of the sample tube prior to sampling

Cultures grown in both bioreactors behaved according to expected growth profiles in terms of antibody production, culture viability, and viable cell counts (Figure 3) and were thus deemed representative of a typical mAb production run.

Samples taken from the two bioreactors were pooled together for each timepoint (once it had been confirmed that cultures from both bioreactors showed comparable measurements for titer, viability and viable cell counts (cf. Figure 3), except on culture day 17 since the culture grown in the second bioreactor (annotated as #2 in the figures and tables) ran out of glucose between day 15 and 17, whereas the culture in the first bioreactor (#1) still had small amounts of glucose left (most likely due to a slight variation in glucose addition on day 7 between the two bioreactors). Consequently, on day 17 the culture viabilities of bioreactors #1 and #2 were 89% and 66% respectively, and the decision was taken to not pool the samples together, but to instead analyze them separately.

### 3.2 Primary recovery

Due to the small volumes of material used in this study (40–50 ml), the pressure during filtration was not measured. As an approximate qualitative measure of filterability, Table 2 compares how many 0.2 μm syringe filters (Mini Kleenpak™ 25 mm syringe filters with 0.2 μm Supor® EKV membrane, Pall Corporation, Portsmouth, UK) were used to filter the material from each sampling day. This shows that material was found to be progressively more difficult to clarify toward the end of cultivation, which could be due to the increasing cell density; additionally, a theory discussed in previous literature^23^ is that apoptotic and nonviable cells suffer a gradual breakdown of the cells' lipid bi‐layer as a result of cell age. Both a loss of membrane integrity as well as an increase in cellular debris is undesirable for the process: an associated release of intracellular impurities would result in less pure product while larger amounts of particulates/cell debris negatively affect the efficiency of the filtration step prior to downstream purification.

**TABLE 2:** Number of 0.2 μm syringe filters (Mini Kleenpak™ 25 mm syringe filters with 0.2 μm Supor® EKV membrane, Pall Corporation, Portsmouth, UK) required to filter HCCF on each sampling day as a rough measurement of filter efficiency, as well as lactate dehydrogenase (LDH) which is used as an indicator of cell lysis due to its intracellular localization

	Viable cell counts (x10^6^ viable cells/ml)	LDH (U/L)	Number of necessary filters
Day 8	13.29	186	1×
Day 10	14.64	250	1×
Day 13	17.78	338	1×
Day 15	17.12	515	2×
Day 17 (#1)	14.57	969	2×
Day 17 (#2)	10.21	2095	3×

### 3.3 Protein A purification

**FIGURE 4:**
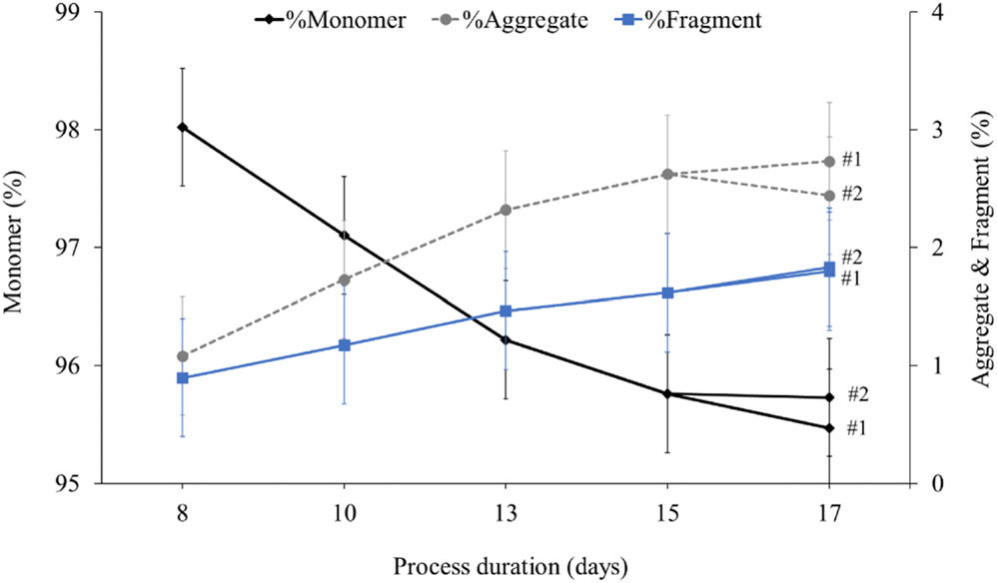
Effects of process duration on product monomer, aggregation, and fragmentation in mAb 1 2 L bioreactor time‐course samples. Data was obtained by SEC following protein A purification. Error bars show ±0.5% assay variability. SEC, size exclusion chromatography

**FIGURE 5:**
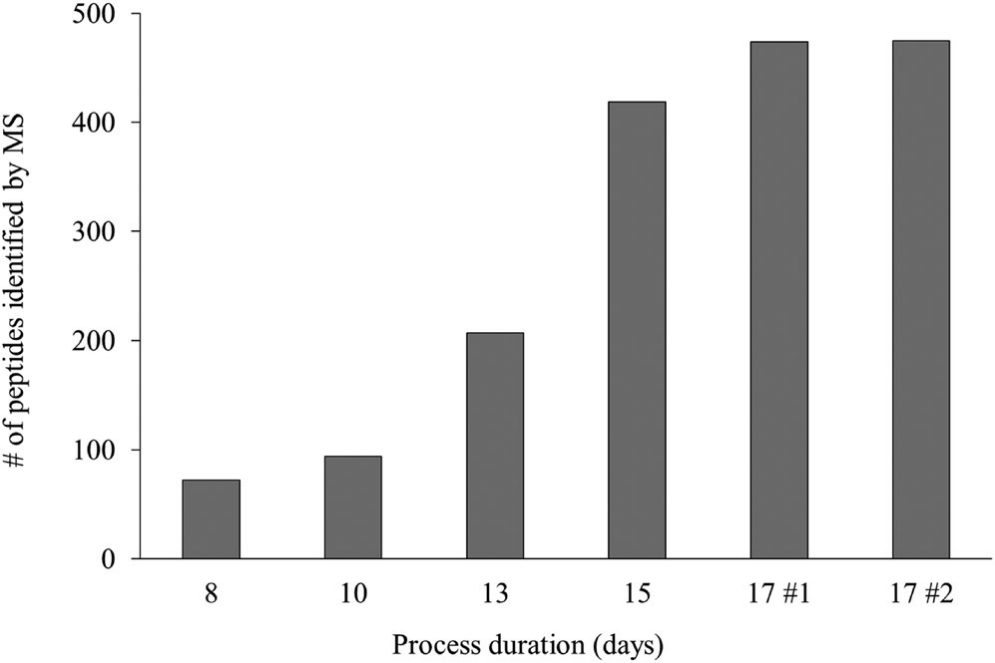
Amount of post‐protein A HCP peptides identified by nano‐LC OrbiTrap in mAb 1 2 L bioreactor cultures on days 8–17. Note that on days 8–15 material from both bioreactors was pooled after confirmation of similar growth and metabolite profiles, whereas on day 17 the cultures from bioreactor #1 and #2 were analyzed separately due to varying viability levels (89% and 66% respectively). During MS data validation, common contaminants as well as HCPs with only two peptides have been filtered out and a MS/MS score of 150 was applied to accept the MS/MS data quality. The remaining data was manually evaluated based on the isotope plot data. HCP, host cell protein; MS, mass spectrometry

Analytical analysis of the protein A purified material showed that product quality decreases in a time‐dependant manner (Figures 4 and 5) which is consistent with previously published literature.^22^

Figure 4 shows that product fragmentation doubles from 0.9% to 1.8% throughout the culture from days 8 to 17 for both bioreactors. Product aggregation steadily increases in bioreactor #1 from 1% to 2.7% while in the material from the second bioreactor, product aggregation was measured to be slightly lower in sample 17 #2 compared to day 15, although when taking the ±0.5% SEC assay variability into account this difference could be negligible. Alternatively, this lower measurement in sample 17 #2 could indicate that product aggregation was consistently higher in the first bioreactor throughout the culture, which might have only been revealed on day 17 when both bioreactors were analyzed separately.

The SEC data shows that product fragmentation and aggregation increases throughout the culture, which could be an indication that cells are struggling to continue protein biosynthesis, including protein folding, or could be caused by cell culture components, HCPs, or inherent product instability.

### 3.4 Mass spectrometry

To gain a deeper understanding of cellular behavior, protein A purified time‐course samples of the cultures grown in the 2 L bioreactors were analyzed by MS and post‐protein A HCP profiles for the cultures were produced. The MS instrument used was a highly sensitive nano‐LC Orbitrap system. The amount of HCP peptides that were identified in each sample of this study are illustrated in Figure 5 and the specific HCP species are listed in Tables 3, 4, 5, 6 where they are grouped by biological function. All biological process information for the identified proteins has been obtained from the UniProt database.^24^ As mentioned before, the risk of false positive results has been reduced by disregarding HCPs with only two peptides and by applying a MS/MS score of 150 to accept the MS/MS data quality. The remaining data was manually evaluated based on the isotope plot data. With regards to missed HCP species, while it is possible that not every single HCP species present within a sample has been detected, the HCP species that have been identified reflect the range of biological processes and pathways that are active.

**TABLE 3:** HCP species involved in glycolysis, lipid metabolism and other carbohydrate metabolisms that were identified by nano‐LC OrbiTrap in mAb 1 2 L bioreactor cultures on days 8–17. Note that on days 8–15 material from both bioreactors was pooled after confirmation of similar growth and metabolite profiles, whereas on day 17 the cultures from bioreactor #1 and #2 were analyzed separately due to varying viability levels. During MS data validation, common contaminants as well as HCPs with only two peptides have been filtered out and a MS/MS score of 150 was applied to accept the MS/MS data quality. The remaining data was manually evaluated based on the isotope plot data

Protein name	Biological process^a^	Location	Day 8	Day 10	Day 13	Day 15	Day 17 #1	Day 17 #2
Glyceraldehyde‐3‐phosphate dehydrogenase	Glycolysis	Intracellular^2^	x	x	x	x	x	x
Pyruvate kinase	Glycolysis	Intracellular^3^	x	x	x	x	x	x
Alpha‐enolase	Glycolytic process	Intracellular^1^		x	x	x	x	x
Phosphoglycerate kinase	Glycolysis	Intracellular^2^				x	x	x
Transketolase	Glyceraldehyde‐3‐phosphate biosynthesis, glycolysis	Intracellular^1^				x	x	
Lipoprotein lipase	Lipid metabolism	Extracellular^1^	x	x	x	x	x	x
Phospholipid transfer protein	Lipid transport	Extracellular^1^	x	x	x	x	x	x
Lysosomal alpha‐glucosidase	Carbohydrate metabolism	Intracellular^2^			x	x	x	x
6‐phosphogluconate dehydrogenase, decarboxylating	Carbohydrate metabolism, pentose phosphate pathway	Intracellular^2^			x	x	x	x
Neutral alpha‐glucosidase AB	Carbohydrate metabolism	Intracellular^2^				x	x	x
UDP‐glucose 6‐dehydrogenase	Carbohydrate metabolism, glycosaminoglycan biosynthesis	Intra‐/extracellular^2^				x	x	x
Malate dehydrogenase	Carbohydrate metabolism, TCA	Intracellular^3^				x	x	x
Tissue alpha‐L‐fucosidase	Carbohydrate metabolism	Intracellular^2^				x	x	
Sialidase‐1	Carbohydrate metabolism	Intracellular^2^				x		

*Note*: Subcellular location information was obtained from (1) UniProt (Chinese hamster data) or (2) UniProt (Human data, where Chinese hamster data was not available) or (3) inferred from biological process data.

^a^ Biological process information was obtained from the UniProt database.^24^

**TABLE 4:** HCP species involved in translation/protein synthesis that were identified by nano‐LC OrbiTrap in mAb 1 2 L bioreactor cultures on days 8–17. Note that on days 8–15 material from both bioreactors was pooled after confirmation of similar growth and metabolite profiles, whereas on day 17 the cultures from bioreactor #1 and #2 were analyzed separately due to varying viability levels. During MS data validation, common contaminants as well as HCPs with only two peptides have been filtered out and a MS/MS score of 150 was applied to accept the MS/MS data quality. The remaining data was manually evaluated based on the isotope plot data

Protein name	Biological process^a^	Location	Day 8	Day 10	Day 13	Day 15	Day 17 #1	Day 17 #2
Elongation factor 1‐alpha	Translation, protein biosynthesis	Intracellular^2^	x	x	x	x	x	x
Elongation factor 1‐gamma	Translation, protein biosynthesis	Intra‐/extracellular^2^			x	x	x	x
Elongation factor 2	Translation, protein biosynthesis	Intracellular^1^			x	x	x	x
40S ribosomal protein SA	Translation, ribosome constituent	Intracellular^1^			x	x	x	x
60S acidic ribosomal protein P0	Translation, ribosome biogenesis	Intracellular^1^				x	x	x
40S ribosomal protein S15a	Translation, ribosome constituent	Intracellular^1^				x	x	x
D‐3‐phosphoglycerate dehydrogenase	L‐serine biosynthesis	Intra‐/extracellular^2^				x	x	x
40S ribosomal protein S16	Translation, ribosome constituent	Intracellular^1^					x	x
Elongation factor 1‐delta	Translation, protein biosynthesis	Intracellular^1^					x	x
C‐1‐tetrahydrofolate synthase, cytoplasmic‐like protein	Amino acid biosynthesis, one‐carbon metabolism, tetrahydrofolate interconversion	Intracellular^2^					x	x
Glycyl‐tRNA synthetase	Translation, aminoacylation of tRNA	Intracellular^1^					x	x
Serine–tRNA ligase, cytoplasmic	Translation, aminoacylation of tRNA	Intracellular^1^						x
Valyl‐tRNA synthetase	Translation, aminoacylation of tRNA	Intracellular^3^						x

*Note*: Subcellular location information was obtained from (1) UniProt (Chinese hamster data) or (2) UniProt (Human data, where Chinese hamster data was not available) or (3) inferred from biological process data.

^a^ Biological process information was obtained from the UniProt database.^24^

**TABLE 5:** HCP species involved in stress responses pathways that were identified by nano‐LC OrbiTrap in mAb 1 2 L bioreactor cultures on days 8–17. Note that on days 8–15 material from both bioreactors was pooled after confirmation of similar growth and metabolite profiles, whereas on day 17 the cultures from bioreactor #1 and #2 were analyzed separately due to varying viability levels. During MS data validation, common contaminants as well as HCPs with only two peptides have been filtered out and a MS/MS score of 150 was applied to accept the MS/MS data quality. The remaining data was manually evaluated based on the isotope plot data

Protein name	Biological process^a^	Location	Day 8	Day 10	Day 13	Day 15	Day 17 #1	Day 17 #2
Clusterin	Chaperone, protein folding	Extracellular^1^	x	x	x	x	x	x
Cathepsin L1	Proteolysis	Extra‐/intracellular^1^	x	x	x	x	x	x
Serine protease HTRA1	Proteolysis	Extra‐/intracellular^2^		x	x	x	x	x
Endoplasmic reticulum chaperone BiP	Chaperone, unfolded protein response	Intracellular^1^			x	x	x	x
Heat shock protein HSP 90‐alpha	Stress response (cytosolic), protein folding	Intracellular^2^			x	x	x	x
Heat shock cognate 71 kDa protein	Stress response (ER), protein folding	Intracellular^2^			x	x	x	x
Hypoxia up‐regulated protein 1	Stress response (ER), cellular response to hypoxia	Intracellular^1^			x	x	x	x
Peroxiredoxin‐1	Stress response to oxidation, cell redox homeostasis	Intracellular^2^			x	x	x	x
T‐complex protein 1 subunit gamma	Chaperone, protein folding, telomere maintenance	Intracellular^1^			x	x	x	x
Glucosylceramidase	Stress response to starvation, lipid glycosylation	Intra‐/extracellular^1^			x	x	x	
Heat shock protein HSP 90‐beta	Stress response (cytosolic), protein folding	Intra‐/extracellular^2^				x	x	x
Heat shock‐related 70 kDa protein 2	Stress response, protein folding	Intracellular^1^				x	x	x
Endoplasmin	Stress response (ER), protein folding	Intracellular^1^				x	x	x
Calreticulin	Chaperone, cellular senescence	Intracellular^1^				x	x	x
T‐complex protein 1 subunit alpha	Chaperone, protein folding, telomere maintenance	Intracellular^1^				x	x	x
T‐complex protein 1 subunit delta	Chaperone, protein folding, telomere maintenance	Intracellular^1^				x	x	x
T‐complex protein 1 subunit theta	Chaperone, protein folding, telomere maintenance	Intracellular^2^				x	x	x
Glutathione S‐transferase P	Stress response, detoxification	Intracellular^2^				x	x	x
Protein disulfide‐isomerase A3	Cell redox homeostasis	Intracellular^2^				x	x	x
Metalloendopeptidase	Proteolysis	Extracellular^1^				x		
Peptidyl‐prolyl cis‐trans isomerase	Protein folding acceleration, cell cycle	Unknown				x		x
T‐complex protein 1 subunit beta	Chaperone, protein folding, telomere maintenance	Intracellular^1^					x	x
T‐complex protein 1 subunit zeta	Chaperone, protein folding, telomere maintenance	Intracellular^1^					x	x
Ubiquitin activating enzyme E1	Ubiquitin activation, proteasome degradation	Intracellular^1^						x

*Note*: Subcellular location information was obtained from (1) UniProt (Chinese hamster data) or (2) UniProt (Human data, where Chinese hamster data was not available).

^a^ Biological process information was obtained from the UniProt database.^24^

**TABLE 6:** HCP species associated with the cytoskeleton that were identified by nano‐LC OrbiTrap in mAb 1 2 L bioreactor cultures on days 8–17. Note that on days 8–15 material from both bioreactors was pooled after confirmation of similar growth and metabolite profiles, whereas on day 17 the cultures from bioreactor #1 and #2 were analyzed separately due to varying viability levels. During MS data validation, common contaminants as well as HCPs with only two peptides have been filtered out and a MS/MS score of 150 was applied to accept the MS/MS data quality. The remaining data was manually evaluated based on the isotope plot data

Protein name	Biological process^a^	Location	Day 8	Day 10	Day 13	Day 15	Day 17 #1	Day 17 #2
Actin, cytoplasmic	Cytoskeleton, cell motility	Intracellular^1^	x	x	x	x	x	x
Procollagen C‐endopeptidase enhancer	Collagen binding	Extracellular^1^		x	x	x	x	x
Tubulin alpha chain	Cytoskeleton, microtubule	Intracellular^1^			x	x	x	x
Tubulin beta chain	Cytoskeleton, microtubule	Intracellular^1^			x	x	x	x
Torsin‐1B	ER organization	Intracellular^2^			x			
Nidogen‐1	Extracellular matrix structural constituent	Extracellular^2^				x		
Procollagen‐lysine,2‐oxoglutarate 5‐dioxygenase 1	Collagen fibril organization, response to hypoxia	Intracellular^2^				x		
Dihydropyrimidinase‐related protein 2	Cytoskeleton organization, axon guidance	Intracellular^1^				x	x	x
Cofilin‐1	Cytoskeleton organization	Intracellular^1^					x	x
Myosin‐9	Cytoskeleton reorganization, cytokinesis	Intracellular^1^					x	x

*Note*: Subcellular location information was obtained from (1) UniProt (Chinese hamster data) or (2) UniProt (Human data, where Chinese hamster data was not available).

^a^ Biological process information was obtained from the UniProt database.^24^

Figure 5 shows that the amount of post‐protein A HCP species increases as culture duration progresses (including a possible rise in specific HCPs that only reach detectable levels by MS toward the end of cell culture). While the focus of this study was on HCP identification by MS and the amount of detected HCP peptides is therefore more qualitative than quantitative, we have previously published the results of a similar data set where HCP levels were measured by ELISA (a more quantitative orthogonal assay) and the trend of increasing HCPs seen here is consistent with the results from the previously published data set.^22^

Figure 5 also reveals that the amount of identified HCP peptides in samples 17 #1 and 17 #2 is very similar, despite the difference in culture viability between the two bioreactors—this will be discussed further below.

As can be seen in Table 3, HCPs present in high amounts include those that are involved in essential cell survival processes such as in crucial glucose or lipid metabolism pathways (e.g., Glyceraldehyde 3‐phosphate dehydrogenase, pyruvate kinase, alpha‐enolase, lipoprotein lipase, phospholipid transfer protein).^24^ These proteins were expected to be highly abundant and were indeed mostly present throughout the entire duration of the culture. Additionally, further carbohydrate metabolism proteins were detected during later stages of the culture (from days 13 and 15), for example, lysosomal alpha‐glucosidase, which is an enzyme usually located in the lysosome rather than the cytosol and could thus be an indicator of cell membrane breakdown.^24^

Further HCPs that were identified are those involved in the crucial cell process of translation (Table 4). Of these, the most abundant protein and one, which also was present from day 8 until harvest, was elongation factor 1‐alpha. Further elongation factor proteins were measured during later stages: elongation factor 1‐gamma and elongation factor 2 (from day 13); elongation factor 1‐delta (day 17/harvest).

Similarly, ribosomal proteins as well as enzymes necessary for aminoacylation of tRNA were detected during later stages of the process, namely 40S ribosomal protein SA (from day 13), 40S ribosomal protein S15a and 60S acidic ribosomal protein P0 (both from day 15), 40S ribosomal protein S16 (day 17); glycyl‐tRNA synthetase, serine‐tRNA ligase and valyl‐tRNA synthetase (day 17).^24^

The fact that these proteins can be measured in HCCF toward the end of the process suggests that cells are producing higher amounts of such proteins during later stages of the culture. Alternatively, or additionally, this could be an indicator of significant cell breakdown, with intracellular proteins being more prevalent in the HCCF at late‐stage culture.

Perhaps most interesting is the detection of HCPs that are commonly produced as a response to stress (Table 5). While extracellularly localized/secreted proteins like clusterin and cathepsin L1 were identified in all samples, regardless of culture duration, other (mostly intracellularly localized) stress‐response proteins were not detected until later. For example, endoplasmic reticulum (ER) chaperone BiP, heat shock protein HSP 90‐alpha, heat shock cognate 71 kDa protein, hypoxia up‐regulated protein 1 (all from day 13 onwards); heat shock protein HSP 90‐beta, heat shock‐related 70 kDa protein 2, endoplasmin, calreticulin, T‐complex protein 1 subunit alpha/delta/theta (all from day 15 onwards); and lastly T‐complex protein 1 subunit beta and zeta (both only on day 17).^17^, ^18^, ^24^ Additionally, ubiquitin activating enzyme E1 was only detected in the second bioreactor on the last day of the process, when culture viability was 66%.

The presence of HCPs such as BiP, endoplasmin, heat shock protein HSP 90‐alpha, heat shock cognate 71 kDa protein and hypoxia up‐regulated protein 1 are strong indicators of stress within the ER, possibly caused by glucose starvation, lack of protein glycosylation, or oxygen deprivation which all lead to the accumulation of unfolded proteins in the ER and in turn to the activation of the unfolded protein response pathway.^25^ The oxidative stress response is further confirmed by the presence of HCPs like peroxiredoxin‐1, glutathione S‐transferase P, protein disulfide‐isomerase A3, and peroxidasin‐like. In summary, the fact that these proteins—which are all involved in chaperoning unfolded proteins, in telomere maintenance, or in proteasomal degradation^18^, ^24^—are accumulating considerably at a later stage in the culture is a strong indication of stress, induced by factors such as cell age. Particularly the presence of proteins involved in telomere maintenance strongly suggests an age‐related impact.

Likewise, the detection of HCPs associated with the cytoskeleton (Table 6) is also an indicator of increased cell membrane porosity and a release of intracellular proteins. While actin was detected from day 8 onward, this HCP is frequently reported in the literature and is known to be highly abundant.^16^, ^26^ However, the detection at late stage culture of cytoskeletal proteins not commonly found in HCCF or processed material suggests a gradual breakdown of cells and release of cytosolic proteins.

**FIGURE 6:**
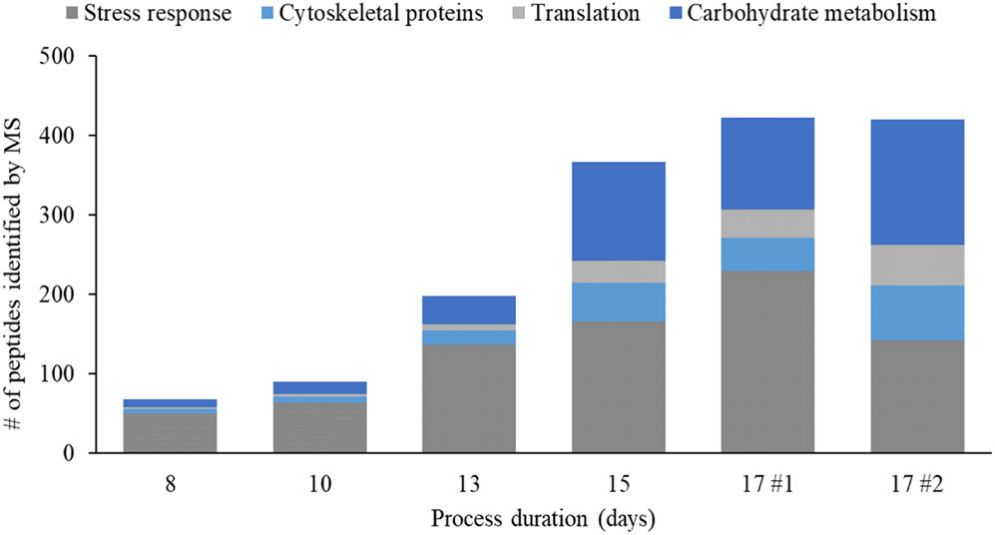
Relative abundance of four groups of HCPs within each sample. HCPs, host cell proteins

**FIGURE 7:**
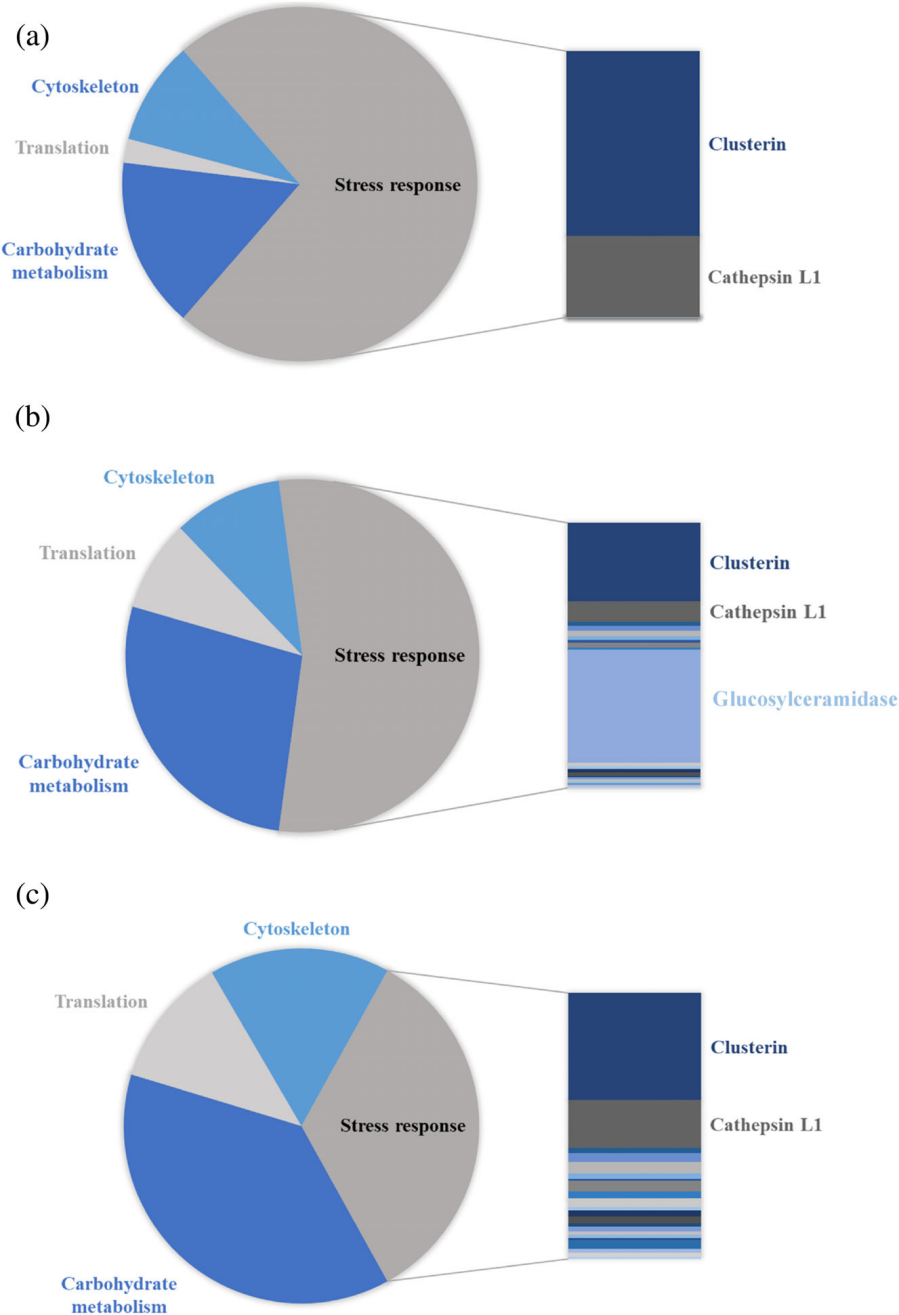
Relative abundance of four groups of HCPs within material from (a) day 8 versus (b) day 17 #1 and (c) day 17 (#2), highlighting the increase in types of stress response proteins. HCPs, host cell proteins

Further to Tables 3, 4, 5, 6, which list the identified HCP species along with the information on which culture days each HCP was detected in protein A purified material, Figure 6 shows the relative abundance of the four discussed groups of HCPs within each sample. Interestingly, on day 8, stress response proteins represent the majority of identified peptides, while they are actually less abundant (relative to the other groups of HCPs) on day 17. However, on day 8, the only stress response proteins that were identified were the highly abundant proteins clusterin and cathepsin L1, whereas on day 17, a total of 21 and 22 proteins involved in stress response pathways (in bioreactors #1 and #2 respectively) were detected (Figure 7; Table 5).

Coming back to the previous observation that the amount of identified HCP peptides in both bioreactors on culture day 17 is very similar (cf. Figures 5, 6, and 7), further reveal that the material from bioreactor #1 contained a higher amount and proportion of stress response proteins relative to bioreactor #2, despite being associated with a higher culture viability. Assuming that cell viability and apoptosis are linked to the release of intracellular enzymes, we would expect to see higher levels of total HCPs as well as presumably higher amounts of stress response proteins in the material from bioreactor #2 due to its lower culture viability.

However, there are several interesting observations to consider here: First, the proportion of intracellular proteins associated with the cytoskeleton, with translation and with carbohydrate metabolism is higher in bioreactor #2 compared to the first bioreactor (cf. Figures 6 and 7), suggesting a greater release of these intracellular proteins, perhaps due to secondary necrosis which can occur after apoptosis.^18^ Second, the detection of ubiquitin activating enzyme E1 in material from bioreactor #2 (cf. Table 5) could be an explanation for the lower than expected levels of host cell proteins, since this enzyme is involved in the ubiquitin proteasome pathway, leading to proteasomal degradation. Last, the previously mentioned declining filter efficiency (cf. Table 2) may have contributed to a small loss of HCPs as the material from sample 17 #2 was more challenging to filter, presumably due to the presence of more cellular debris, which might have resulted in a possible removal of proteins during filtration.

Reiterating the previously mentioned theory that apoptotic and nonviable cells suffer a gradual breakdown of cells' lipid bi‐layer as a result of cell age resulting in increased porosity of the membrane and a loss of membrane integrity,^23^ it can therefore be concluded that the MS data presented here supports this hypothesis as several HCP species were detected which are indicators of cell age and cellular membrane breakdown.

### 3.5 Previous literature

Some of the proteins presented here have also been identified in previous literature^16^, ^17^, ^18^, ^27^ although HCP identification data has not commonly been presented in relation to culture duration and biological processes (Table 7).

**TABLE 7:** Selection of published literature that contains HCP species identification data, along with an overview of the type of analyzed samples, and whether results were linked to culture duration or biological processes

Literature	Samples	Related to culture duration?	Related to biological process?
Albrecht et al., 2018^18^	HCCF	No	Yes
Farrell et al., 2015^17^	Post‐protein A	Yes (days 5 and 7)	No
Zhang et al., 2014^16^	HCCF Post‐protein A Post‐viral inactivation Post‐ion exchange	No	No
Zhang et al., 2016^27^	Post‐protein A	No	No

Abbreviation: HCCF, harvested cell culture fluid.

Albrecht et al.^18^ have carried out MS to study HCP profile changes during cell stress and cell death using apoptosis and necrosis models. HCPs were measured in HCCF rather than in protein A purified material, but several of the species they identified have also been detected here in late stage culture material, that is, they have been carried over during protein A purification, for example, heat shock cognate 71 kDa protein and heat shock protein HSP 90‐alpha (detected from day 13 onward), endoplasmin, glutathione S‐transferase P, and heat shock protein HSP 90‐beta (detected from day 15 onward), and cofilin‐1 (detected on day 17).

Farrell et al.^17^ have used MS to determine post‐protein A HCP profiles as a function of culture harvest time—although only comparing day 5 (the start of the stationary phase) and day 7 (the end of the stationary phase). They found that product which is harvested at the later stage of cell culture contained higher concentrations of HCPs. Furthermore, the HCPs identified on day 5 were mainly secreted proteins (such as clusterin and procollagen C‐endopeptidase enhancer), whereas most HCPs (>70%) identified on day 7 were intracellular proteins (e.g., 78 kDa glucose‐regulated protein, calreticulin, glyceraldehyde‐3‐phosphate dehydrogenase, histone H2AX, and serine protease HTRA1). They theorized this was likely due to cell lysis rather than increased secretion of proteins. The results from Farrell et al.^17^ are congruent with the data presented in this article; however, the culture duration investigated here is far more extensive than the one studied by Farrell et al.^17^

Zhang et al.^16^ have used MS to track HCP species from HCCF through the downstream purification steps: protein A purification, viral inactivation, and polishing chromatography. They used nine mAbs for the study and have published approximately 40 identified HCP species, many of which have also been detected and presented here.

In another publication,^27^ this research group has further compared post‐protein A HCP profiles among 15 different mAbs and found that on average only 10% of post‐protein A HCPs were specific for each individual mAb, while the remaining post‐protein A HCPs were common to all mAbs. HCPs that were common to all investigated mAbs were, for example, clusterin, actin, elongation factor 1 alpha 1, heat shock cognate 71 kDa protein, 78 kDa glucose regulated protein, glyceraldehyde‐3‐phosphate dehydrogenase, glutathione S transferase P and serine protease HTRA1.

Unfortunately, Zhang et al. did not specify in either of their publications^16^, ^27^ how long their cultures were maintained for and on which days material was harvested, so it is impossible to link the published HCP species to a particular harvest timepoint, and fully compare the data to the results presented here, where an extensive culture duration context is provided, nor did they assign the HCPs to the biological processes that they are involved in, which would provide insight into cells' behavior during mAb production.

In conclusion, information about the specific HCPs that co‐purify with mAbs during protein A chromatography is progressively increasing with each new published dataset. Understanding the mechanism by which HCPs are retained during protein A purification is crucial to enable the development of a targeted HCP clearance strategy. The identification of HCP species presented here provides a new level of insight into HCPs that are retained during mAb purification which can be applied to increase our understanding of cellular behavior during production of therapeutic antibodies as well as to design targeted HCP clearance strategies during protein A purification, both of which may be used to aid process development strategies.

## 4 CONCLUSIONS

To investigate the impact of cell culture duration on critical quality attributes, a CHO‐expressed IgG1 was cultivated in two 2 L bioreactors and samples were taken on days 8, 10, 13, 15, and 17. The material was centrifuged, filtered and protein A purified on a 1 ml HiTrap column. It was shown that as cultivation progressed and antibody titer increased, product monomer levels steadily decreased while post protein A HCP impurities increased, indicating that harvest material is becoming progressively more difficult to recover using this purification scheme, and that culture duration should not be extended purely for the purpose of expressing more antibody.

Furthermore, HCP identification by MS was performed on material from different timepoints to provide insights into cellular behavior and HCP carryover during protein A purification. The data showed increases in several classes of post‐protein A HCPs (e.g., HCPs involved in carbohydrate metabolism, cytoskeletal proteins, and stress response proteins), particularly on days 15 and 17 of culture, which were associated with significant increases in total HCP levels. The HCP species identification confirmed a previously published theory that apoptotic and nonviable cells suffer a gradual breakdown of cells' lipid bi‐layer as a result of cell age resulting in increased porosity of the membrane and a loss of membrane integrity,^23^ since several HCP species were detected here which are indicators of cell age and cellular membrane breakdown.

One thing to note is that the trends seen in this article are based on the results of post‐protein A analysis, and the HCPs present in protein A eluate are extrapolated to provide insight with respect to the state of the growing cells. As previous literature^2^, ^10^, ^11^, ^12^, ^13^, ^14^ has demonstrated only specific subpopulations of HCPs co‐elute with the antibody during protein A affinity chromatography while others are cleared during this purification step. However, while more HCP species may have been present in pre‐protein A material, the HCP species that have been identified here very likely still reflect the range of biological processes and pathways that are active.

## 5 FUTURE WORK

Host cell protein identification by MS is incredibly valuable and enables a combination of a process engineering approach with a strong biochemical analysis of the identified HCP species present under various process conditions. This serves several purposes. Such research will help identify process conditions resulting in product which is associated with HCP species that are known to be a safety risk to patients and thus help avoid growing cells in such conditions. It can also greatly enhance our understanding of the cells we use to synthesize therapeutic proteins. Identifying the proteins that host cells produce under different growth or stress conditions and at different times during culture enables the use of biochemical analysis to better understand cellular behavior, for example, which metabolic pathways are active; are cells overstrained and activating the unfolded protein response pathway or even stressed to the point that apoptotic pathways are being activated. This level of understanding will benefit attempts to influence cellular behavior and optimize conditions for high production of good quality therapeutic proteins.

One possible way this could be done is by understanding the factors that lead to apoptosis and the apoptotic pathways that are activated and then exploring ways to prevent or counter‐act the activation/progression of these pathways. A similar approach could be applied to the identification of proteins involved in stress response pathways, like chaperones involved in the unfolded protein response pathway, which could be used as early indicators of cellular stress.

Another strong benefit of HCP profile characterization by MS is the identification of HCP species which are particularly problematic to remove from the final drug product and which are known to compromise patient safety. Being aware of such proteins facilitates attempts to prevent their production altogether by genetic engineering of the gene in question.

Most importantly, this research will establish a base understanding of the cells used in the biopharmaceutical industry. This will be crucial as technological advances will likely lead to significant changes in upstream and downstream processing, such as a switch from fed‐batch to perfusion culture in upstream, and multi‐column continuous chromatography or new resins in downstream. These issues will be compounded with a move towards generally more complex therapeutic protein structures as opposed to relatively well‐established monoclonal antibody structures.

## CONFLICT OF INTEREST

The authors declare no conflict of interest.

## AUTHOR CONTRIBUTIONS

**Louisa J Wilson:** Data curation (lead); formal analysis (lead); visualization (equal); writing – original draft (lead); writing – review and editing (equal). **William J Lewis:** Conceptualization (equal); funding acquisition (equal); resources (equal); supervision (equal); visualization (equal); writing – review and editing (equal). **Richard Kucia‐Tran:** Methodology (equal); supervision (equal); visualization (equal); writing – review and editing (equal). **Daniel Gilbert Bracewell:** Conceptualization (lead); funding acquisition (lead); supervision (lead); writing – review and editing (equal).

### PEER REVIEW

The peer review history for this article is available at https://publons.com/publon/10.1002/btpr.3224.

## Data Availability

The data that support the findings of this study are available from the corresponding author upon reasonable request.
